# Language Translation Apps in Health Care Settings: Expert Opinion

**DOI:** 10.2196/11316

**Published:** 2019-04-09

**Authors:** Anita Panayiotou, Anastasia Gardner, Sue Williams, Emiliano Zucchi, Monita Mascitti-Meuter, Anita MY Goh, Emily You, Terence WH Chong, Dina Logiudice, Xiaoping Lin, Betty Haralambous, Frances Batchelor

**Affiliations:** 1 National Ageing Research Institute Parkville Australia; 2 Northern Health Epping Australia; 3 St Vincent's Hospital Fitzroy Australia; 4 National Ageing Research Institute , Parkville, VIC Academic Unit for Psychiatry of Old Age, University of Melbourne Melbourne Health, Parkville VIC Parkville Australia; 5 Academic Unit for Psychiatry of Old Age University of Melbourne Parkville Australia; 6 Melbourne Health Parkville Australia

**Keywords:** health care, communication, language, technology

## Abstract

**Background:**

Currently, over 300 languages are spoken in Australian homes. People without proficient English from non-English speaking countries may not receive equitable care if their health care workers do not speak their primary language. Use of professional interpreters is considered the gold standard; however, for a variety of reasons, it is often limited to key aspects of care such as diagnosis and consent. With the emergence of mobile technologies, health care workers are increasingly using digital translation tools to fill this gap. However, many of these technologies have not been developed for health care settings and their use has not been evaluated.

**Objective:**

This study aimed to evaluate iPad-compatible language translation apps to determine their suitability for enabling everyday conversations in health care settings.

**Methods:**

Translation apps were identified by searching the Apple iTunes Store and published and grey literature. Criteria for inclusion were that the apps were available at no cost, able to translate at least one of the top 10 languages spoken in Australia, and available for use on iPad. Apps that met inclusion criteria were reviewed in 2 stages. Stage 1 was the feature analysis conducted by 2 independent researchers, where apps were evaluated for offline use, input and output methods, and number of languages. Stage 2 was the analysis of suitability for everyday communication in the health care setting, conducted by 2 independent professionals with expertise in translation and cross-cultural communication. Apps that enabled key aspects of care normally within the realm of professional interpreters, such as assessment, treatment and discharge planning, and seeking consent for medical treatments, were considered unsuitable.

**Results:**

In total, 15 apps were evaluated. Of these, 8 apps contained voice-to-voice and voice-to-text translation options. In addition, 6 apps were restricted to using preset health phrases, whereas 1 app used a combination of free input and preset phrases. However, 5 apps were excluded before stage 2. In addition, 6 of the 10 remaining apps reviewed in stage 2 were specifically designed for health care translation purposes. Of these, 2 apps were rated as suitable for everyday communication in the health care setting—culturally and linguistically diverse Assist and Talk To Me. Both apps contained simple and appropriate preset health phrases and did not contain conversations that are normally within the realm of professional interpreters.

**Conclusions:**

All iPad-compatible translation apps require a degree of caution and consideration when used in health care settings, and none should replace professional interpreters. However, some apps may be suitable for everyday conversations, such as those that enable preset phrases to be translated on subject matters that do not require a professional interpreter. Further research into the use of translation technology for these types of conversations is needed.

## Introduction

### Background

The widespread prevalence of telemedicine and telehealth has led to an increasing acceptance of technology in health care. Although there is limited evidence for the effective use of translation technology in medical and health care settings, clinicians anecdotally report the use of internet and mobile apps for language translation purposes. This raises potential concerns as most apps have not been specifically developed or validated for use in a medical or health care context. However, there is potential for technology to be used to improve everyday clinical communication between patients and staff in the health care setting when used as an adjunct to professional interpreters [1].

Australia is one of the most ethnically and culturally diverse countries in the world [2]. According to the Australian Bureau of Statistics [2], almost half of all Australians were either born overseas or had at least one parent who was born overseas. In 2015, Australia had the ninth largest population of people born overseas worldwide and a higher proportion of overseas-born people (26%) compared with other countries founded on migration such as New Zealand (23%), Canada (22%), and the United States (14%) [2]. Net overseas migration continues to increase in Australia and has shown periods of influx linked to major world events. For example, following the Second World War, Australia saw a high proportion of European migrants. In more recent times, migration has predominantly been from China, India, and the Middle East, with Asian countries now making up 8 out of the top 10 migration countries to Australia [3]. In 2016, there were over 300 different languages spoken in Australian homes and more than one-fifth of Australians spoke a language other than English at home [2]. After English, the 10 most common languages spoken at home in Australia are Mandarin, Arabic, Cantonese, Vietnamese, Italian, Greek, Hindi, Spanish, Punjabi, and Tagalog [2].

The ability to convey essential care needs (eg, addressing pain, help with hygiene), communicate simple safety messages, and provide orientation cues are essential in health care settings. People without proficient English from non-English speaking (NES) countries may not receive equitable care if their health care workers do not speak their primary language [4,5]. The use of professional interpreters is considered the gold standard [6]. However, because of issues related to cost, access, availability, and time constraints [7,8], use of professional interpreters in health care is often limited to specific aspects of care, such as comprehensive assessments, procedural consent, diagnosis, and the development of treatment plans. Everyday communication between health care workers and clients, when there is a language barrier, generally occurs without professional interpreters and has been described in the literature as *getting by*, where health care workers rely on gestures, facial expressions, and knowledge of minimal key words in the target language [9,10]. The *getting by* approach has the potential for miscommunication, which may lead to inappropriate or inadequate care provision and patients’ needs being unmet. At worst, the *getting by* approach can result in the provision of inappropriate or nonbeneficial treatments and care as highlighted by Runci et al (2012) [11], finding a higher frequency of prescription of antipsychotic drugs for Italian speaking residents in English-speaking residential care facilities than their counterparts in language specific facilities.

Although using an interpreter remains the gold standard for complex medical and legal discussions in all settings, in some situations, it is not appropriate or feasible to use an interpreter, yet communication remains an issue. Through the widespread uptake of mobile devices, technology enabling language translation has been identified as a potential way to improve communication between patients and staff in health care settings when used as an adjunct to professional interpreters [1]. Very few studies have evaluated the use of translation apps in medical and health care settings and even fewer have compared multiple translation apps or examined the contexts in which their use may be suitable.

Although early studies of Web-based language tools, such as Google Translate, highlight high levels of user satisfaction [12], the risks relating to accuracy when used in the clinical setting have become more apparent [12-15]. One study evaluated text translation of 10 common questions relating to medical history and assessment from English into 10 languages and found a wide discrepancy in the accuracy depending on the target language [13]. Vietnamese and Polish translation had the lowest accuracy (10% correctly translated), whereas Spanish had the highest accuracy (80% correctly translated). Another study evaluated the accuracy of 10 medical phrases in 26 languages [14]. Of the total translations, approximately 58% of the translations were accurately translated from English. However, the accuracy among the different languages also showed variability, with African languages scoring the lowest accuracy (42%), followed by Asian languages (46%), then Eastern European languages (62%), and Western European languages (74%). The authors reported the presence of some phrases where the translation resulted in considerable changes to the intended meaning when using complex medical terminology in high-risk situations. For example, “Your child is fitting” was incorrectly translated to “Your child is dead” in Swahili and “Your husband has the opportunity to donate his organs” was incorrectly translated to “Your husband can donate his tools” in Polish. As a result, the authors cautioned against the use of Google Translate when obtaining consent for surgery or other medical procedures and participation in research.

Beh and Canty [15] also reviewed the accuracy of Google Translate in a simulated preanesthetic consultation between an English-speaking anesthetist and a Mandarin-speaking patient. In total, 24 English phrases and 13 Mandarin phrases were tested and an independent anesthetist fluent in both English and Mandarin assessed the translation accuracy. The accuracy of translation from English to Mandarin was 72% and from Mandarin to English was 67%, improving with short or simple phrases that did not contain technical information or when speaking clearly and slowly. The authors concluded that Google Translate was not accurate enough to replace professional interpreters but might be useful if an interpreter was not available.

Albrecht et al [16] conducted a 6-week trial of a German translation app called *xprompt–multilingual assistance*, designed for use in health care settings. The app allowed for preset health phrases to be translated and was used for basic conversations. Nursing staff were then surveyed about their experiences. Although most staff reported that the translation tool was helpful for communicating with patients who spoke another language, was easy to use, and that there were no obvious problems with the usability of the device, some reported that the technology was not practical, was too time consuming, and did not integrate well into existing workflows. They also reported difficulties using the technology with older patients who were unfamiliar with technology or unable to use the app because of visual impairment or illiteracy. The staff also reported that the desired target language was not always available. Explaining the menu items in the app caused problems in some instances.

### Objectives

Given the availability and widespread acceptance of language translation apps by the general public and anecdotal evidence of their use in health care settings, more research is required to evaluate their use, particularly in health care situations when professional interpreters are not normally used, such as everyday clinical conversations, and with particular cohorts, such as older people from NES backgrounds. To date, research evaluating language translation technology in health care settings has done so in complex situations, such as those that involve seeking consent [14], conducting medical assessments [13], or engaging in technical or complex medical conversations [15].

A previous study [17] evaluated mobile medical translation apps where the authors identified key features and scored each app on the basis of usability and functionality. This evaluation aims to provide an overview of iPad (Apple Inc) compatible language translation apps (at no cost) and considerations for use in real-world health care settings. This study uses experts in the field of health care translation and cross-cultural communication to evaluate the content of translation apps and provide expert opinion regarding their suitability for health care situations in which an interpreter would not be available, such as providing orientation cues and conveying essential care needs, including identifying pain or the need for toileting. This is the first study to evaluate translation apps on the basis of their suitability for everyday conversations in the health care setting.

## Methods

### Study Design

The study design involves 2 components:

1. A search for iPad-compatible language translation apps at no cost.
2. An evaluation of retrieved apps comprising 2 stages—feature analysis and analysis of suitability for everyday clinical conversations in health care settings.

### Component 1: Search for Available Language Translation Apps

**Figure 1:**
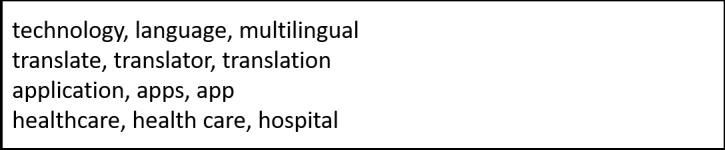
Search Terms for Apple Store.

**Figure 2:**
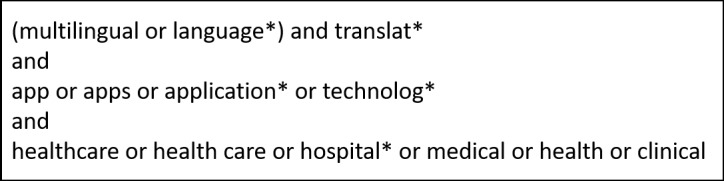
Search Terms for Literature, iMedicalApps and Google Search.

Searching for iPad-compatible translation apps was conducted by first searching the Apple iTunes Store (Apple Inc) on 22 August, 2017, using the search terms in Figure 1. Following this, grey literature (Google Search and Google Scholar) and published literature (PubMed) searches were conducted for published articles related to smartphone or tablet apps used in health care settings for translation purposes. Later, iMedicalApps, a website that reviews all medical apps, was searched using the terms in Figure 2. Finally, any apps that the authors were familiar with from professional experience, which were not discovered in the previous searches, were included.

Translation apps were included if they were developed for or used for language translation purposes, were available at no cost, were available on iPad (Apple Inc), and enabled translation to or from English. Each app had to include translation to at least one of the top 10 languages spoken in Australian homes as of 2016 (Mandarin, Arabic, Cantonese, Vietnamese, Italian, Greek, Hindi, Spanish, Punjabi, and Tagalog, excluding English) [2]. The apps had to operate on iOS 10.3 that was available during August 2017 on the iPad. Apps that required a fee or were only available for use on Android devices were excluded as this project forms a part of a larger pragmatic evaluation of the use of iPad-compatible apps in health care settings, where budgetary limitations are known to prohibit health care providers use of paid apps.

### Component 2: Evaluation of Retrieved Apps

#### Stage 1: Feature Analysis

Once identified, an analysis of all apps that met inclusion criteria was conducted by 2 independent clinical researchers (AP and RTJ). The researchers both had clinical backgrounds and extensive knowledge of technology. The researchers evaluated the apps according to the following key categories: offline use, input and output method, and languages available. Issues that may arise for use in the health care setting were also recorded, including ease of use on the basis of whether the app required a high level of user knowledge or required many steps to navigate through the app (Multimedia Appendix 1). A consensus approach was adopted by the 2 clinical researchers on all aspects of each app. Apps were excluded after the feature analysis stage if they required any in-app purchases or subscriptions, as this was considered a barrier to use in the health care setting as part of a larger study.

#### Stage 2: Analysis of Suitability for Everyday Communication

The apps included were then evaluated on the basis of their suitability for everyday communication in health care settings by 2 independent professionals (EZ and MM) with expertise in translation and cross-cultural communication in health care. Both experts are academically qualified in language and cultural studies, are polyglots, and have extensive experience as language and cultural diversity managers in large public hospitals. This stage of the evaluation focused on the suitability of apps for situations in which an interpreter would not be necessary, such as providing orientation cues and conveying essential care needs, including identifying pain or the need for toileting. Apps were considered less suitable when they contained content or allowed for conversations that were considered critical points in health care. These critical conversations were defined, in line with the state government Language Services Policy [18], as those involving clinical assessment, provision of diagnoses, conversations about treatment and care planning, discharge planning, and medicolegal information such as seeking consent for medical treatments. These types of conversations were considered beyond the scope of translation technology as they require a professional interpreter because of the need for a high degree of accuracy, the need to confirm understanding from patients, and the need to allow patients to ask questions. Other factors that could have an impact on suitability for everyday communication in health care settings were also identified. These included the type, content, and structure of phrases available for selection in the apps, taking into consideration the complexity and sensitivity of information and the ability to allow open-ended or 2-way conversation with appropriate responses available for selection by the patient. Recommendations for use in a health care setting were included in this stage (see Table 1). The evaluators were required to reach consensus about each aspect of each app.

**Table 1:** Analysis of suitability for everyday conversations in the health care setting.

App name	Can the app be used for critical points in health care requiring professional interpreters					Other factors or comments	Overall rating of suitability for everyday communication (low or high)
	Ax^a^	Dx^b^	Tx^c^	D/C^d^	Medicolegal^e^		
CALD^f^ Assist	Y^g^	N^h^	Y	Y	N	Some phrases and questions are lengthy or complex. Only translates preset phrases. The phrases relating to assessment, treatment, and discharge are considered within the scope of everyday clinical conversations (eg, “Do you have pain?”; “I need to do a scan of your bladder”; “You are leaving hospital today”).	High
Canopy Speak	Y	Y	Y	Y	Y	Many questions are lengthy, highly detailed, complex, open-ended, cover a broad scope, and are highly sensitive (eg, “Do you have thoughts of killing others?”; “When you take that medicine, does it make you feel sleepy, give you a headache, or make you feel nauseated?”; “Do you use tobacco now? In the past? For how long? Type and amount daily?”).	Low
Google Translate	Y	Y	Y	Y	Y	Free input allows for any information input to be translated. Therefore, it is considered beyond the scope of everyday clinical conversation.	Low
MediBabble Translator	Y	N	N	N	Y	Many questions are lengthy, highly detailed, complex, sensitive, and/or cover a broad scope, which is beyond the scope of everyday clinical conversation (eg, “Are you allergic to any medication?”; “Do you think about harming yourself?”; ”I’d like to know what the pain feels like”; “Do you experience recurrent or persistent thoughts, impulses, or images that are inappropriate or upsetting?”; “Are you experiencing prolonged or excessive menstrual bleeding at irregular intervals or more frequently than your normal menstrual periods?”).	Low
Microsoft Translator	Y	Y	Y	Y	Y	Free input allows for any information input to be translated. Therefore, it is considered beyond the scope of everyday clinical conversation.	Low
Naver Papago Translate	Y	Y	Y	Y	Y	Free input allows for any information input to be translated. Therefore, it is considered beyond the scope of everyday clinical conversation.	Low
SayHi Translate	Y	Y	Y	Y	Y	Free input allows for any information input to be translated. Therefore, it is considered beyond the scope of everyday clinical conversation.	Low
Talk To Me	Y	N	Y	Y	N	Only translates preset phrases. The phrases relating to assessment, treatment, and discharge are considered within the scope of everyday clinical conversation (eg, “Are you sad?”; “I will take your blood pressure”).	High
TripLingo	Y	Y	Y	Y	Y	Free input allows for any information input to be translated. Therefore, it is considered beyond the scope of everyday clinical conversation.	Low
Universal Doctor Speaker	Y	Y	Y	Y	U^i^	Only allows for limited preset phrases and questions to be translated. In addition, includes open-ended questions, which are considered beyond the scope of everyday clinical conversation. Medical information about an individual can be saved and this poses a risk to confidentiality (eg, “Are you allergic to any medication?”; “You have the following illness or condition—depression / anxiety / chronic depression / obsessive-compulsive disorder, etc”).	Low

^a^Ax: assessment.

^b^Dx: diagnosis.

^c^Tx: treatment and care planning.

^d^D/C: discharge.

^e^medicolegal: medicolegal conversations including consent.

^f^CALD: culturally and linguistically diverse.

^g^Y: yes.

^h^N: no.

^i^U: unsure.

## Results

**Figure 3:**
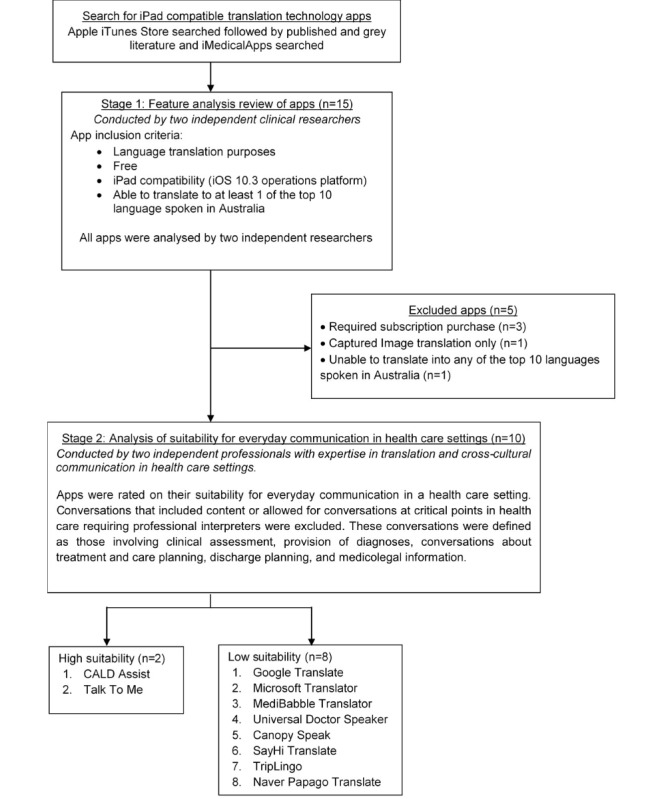
Flow diagram of the process used to identify eligible apps for languages translation. CALD: culturally and linguistically diverse.

In total, 15 apps met the inclusion criteria and were evaluated (Figure 3).

### Stage 1: Feature Analysis

An initial agreement of 93% (70/75) was achieved among the researchers regarding the features available within each app. Differences identified on the remaining aspects of an app were resolved via discussion. Most apps enabled free voice or text input, and this feature usually required an internet connection even after the language package had been downloaded. In total, 53% (8/15) of the apps were capable of voice-to-voice translation, 53% (8/15) of the apps were capable of voice-to-text translation, 33% (5/15) of the apps were capable of text-to-voice translation, and 33% (5/15) of the apps were capable of text-to-text translation. In total, 7 out of 15 apps (47%) enabled the translation of preset phrases. However, 6 of these 7 apps did not allow for free input. Furthermore, 33% (5/15) of the apps could be used offline, but they required language packages to be downloaded. Although TripLingo was capable of multiple input and output functions, it was not specifically designed for the health care setting and contained very few preset phrases that were considered suitable.

In addition, 6 of the 15 apps (40%) were related specifically to health care translation. These were CALD Assist, Canopy Speak, Dr. Passport (Personal), MediBabble Translator, Talk To Me, and Universal Doctor Speaker. All of these apps were limited to the use of preset phrases and did not allow free voice, text, or image input. Only 2 of the 15 apps (13%) were capable of 2-way conversation between a patient and health care worker—CALD Assist and Dr. Passport. In addition to containing closed questions that required a simple *Yes* or *No* response, CALD Assist also enabled some open-ended questions with limited selections to be made by the patient and some follow-up questions. An example of a follow-up question was “Have you lost weight in the last six months?” then “How much weight have you lost?” Several options were available on the screen for the patient to select. Dr. Passport also allowed for 2-way conversation between a patient and health care worker. However, this was only possible by enabling patients to select preset phrases to translate to their health care worker. This app is divided into preset health phrases for the patient and a separate section for health care workers. This app appeared to be intended for patient-led conversations and not for conversations led by health care practitioners.

Of the 15 apps evaluated in stage 1, only 10 continued to stage 2 (Figure 3). iTranslate, iTranslate Voice, and Speak and Translate were excluded as they required monthly subscriptions once the free trial period had ended. Waygo was excluded as it only translated captured images (ie, text within images). Dr. Passport was also excluded as it was only available for free when using it to translate from English to Japanese. All other language translations required a fee.

### Stage 2: Analysis of Suitability for Everyday Communication in Health Care Settings

An initial agreement of 73% (44/60) was achieved between the evaluators regarding the evaluation of the content of the translation apps. Differences identified on the remaining aspects of an app were resolved via discussion.

Of the 10 apps evaluated for their suitability, none were entirely suitable (refer to Table 1). All 10 apps enabled conversations about assessment and all apps, except for one (MediBabble Translator), enabled conversations about treatment or care planning and discharge. Furthermore, 3 of the 10 apps did not enable conversations about diagnosis and medicolegal information. The apps that enabled conversations in the least number of critical points in health care were MediBabble Translator, CALD Assist, and Talk To Me. This contributed to an overall suitability rating of either high or low suitability. The 2 apps, CALD Assist and Talk To Me, were rated as highly suitable on this basis.

## Discussion

### Principal Findings

This study evaluated iPad-compatible language translation apps on the basis of their features and provided expert opinion on their suitability for everyday conversations in which an interpreter would not be available, such as providing orientation cues and conveying essential care needs, including identifying pain or the need for toileting, in a real-world health care setting. This is the first study to involve experts in health care translation and cross-cultural communication in the evaluation of translation technology suitability. This study’s results show that only 2 apps could be considered suitable.

In total, 15 iPad-compatible language translation apps were identified from searches in the Apple iTunes Store and published and grey literature that met the inclusion criteria. These apps were evaluated in 2 stages to analyze features and suitability for everyday communication in health care settings. The feature analysis stage identified that the apps enabled translation of over 100 languages, enabled different input and output modes, which determined whether they could be used offline, and had been developed for a range of different purposes, most commonly for health care, travel, and business purposes. Apps that had been designed for other purposes, such as travel or business, were considered to have limited applicability to the health care setting. Of these 15 apps, 5 were excluded from stage 1 and 10 apps were then evaluated for their suitability of enabling everyday conversations in a health care setting. All apps evaluated in stage 2 required a degree of caution and consideration before being used in health care settings. However, the degree of suitability varied across apps. For example, apps that allowed free input of information to be translated were deemed less suitable as there were no limits to the way the apps could be used, whereas apps that only enabled translation of preset phrases had the potential to limit the contexts in which the apps were used and were deemed more suitable. Despite this, all the apps enabled conversations about topics considered to be critical points in health care, which would normally require professional interpreters, such as clinical assessment and conversations about treatment and care planning. Translating these conversations requires a high degree of accuracy and the ability to confirm understanding from patients and allow patients to ask questions, which are not met by these translation apps. Discussing these topics with translation technology could result in miscommunication, which might lead to serious negative health outcomes for patients.

Apps considered to be most suitable for the health care setting were CALD Assist and Talk To Me. Both enabled conversations in the least number of critical phase topics that required professional interpreters and limited the translation to preset phrases that were led by health care practitioners. Although MediBabble Translator limited topics of discussion to 2 critical phase topics and restricted input to preset phrases, the phrases were deemed to be outside the scope of everyday clinical conversations as they included highly detailed, sensitive and/or personal open-ended questions, such as “Do you think about harming yourself or others?”

A recent study [17] evaluated mobile medical translation apps where the authors identified key features and scored each app on the basis of usability and functionality. Apps that were low cost, able to be used offline, and did not contain advertisements and in-app purchases were compatible with multiple platforms (iPhone, iPad, Android, and Nexus), were easy to navigate, and were well presented, and these apps scored highly. The study rated Canopy Medical Translator, Universal Doctor Speaker, and Vocre Translate favorably. However, these apps were either excluded at stage 1 of our evaluation on the basis of requiring payment or were rated as beyond the scope of everyday clinical conversations at stage 2. Given that this study focused on the clinical utility of translation apps for enabling everyday conversations in the health care setting and that eligibility for inclusion was determined on the basis of informing a larger pragmatic evaluation, which necessitated that the apps were available at no cost and were compatible with iPads, the ratings differed.

### Considerations for Clinical Use

This study evaluated translation technology features and provided expert opinion regarding their suitability for everyday clinical conversations, not necessitating professional interpreters. These factors are important considerations for use in health care settings and for the development of new translation technologies. In addition to these considerations, other factors such as current policy regarding the use of translation technology, data security, and confidentiality should be considered carefully in the design and use of apps for health care. Those that require access to the internet or that keep a record of conversations may require additional precautions, for example, not disclosing the patient’s identity with the app, avoiding collection of personal or sensitive information with the app, avoiding the use of personal devices when using Web-based apps, and using a secure internet connection so that the individual device or location cannot be identified. Other considerations in design and development of apps may include avoiding use of a device with a small screen (eg, smartphone), which may pose difficulties for patients with visual impairments or reduced motor dexterity.

It is not possible to provide access to professional interpreters for a patient’s entire health care episode, and there is an urgent need for effective, accessible, and safe tools, such as translation technology, to facilitate everyday communication to improve health outcomes. However, there is a risk that translation technology may become the preferred means of communicating with patients with limited English proficiency because of the perceived simplicity, accessibility, and timeliness. Over dependence and over reliance on this technology may impact negatively on establishing rapport and providing high quality care. CALD Assist and Talk To Me were the only apps included in this evaluation that provided users with a disclaimer about their limitations and stressed the importance of using professional interpreters where possible. CALD Assist and Talk To Me were specifically designed for health care settings and both restricted communication to preset phrases that were considered within the scope of everyday clinical conversations. They did not include topics and situations that were considered to require professional interpreters. Although Canopy Speak and MediBabble Translate were also specifically designed for health care settings, the evaluators found that these apps were difficult to navigate and contained content that was beyond everyday clinical conversations, requiring a professional interpreter.

### Limitations

It was beyond the scope of this study to examine translation accuracy and cultural suitability. These are important aspects of apps that would have an impact upon the effectiveness of use in health care settings and involve the suitability of translated words for the context, the syntax of the translated phrases (eg, order of words and grammar), and the ability to recognize different accents and dialects (when using free voice input). Previous studies have identified poorer accuracy for the translation of non-western languages in Google Translate [14-16]. Further research involving experts, health professionals, and consumers is required to evaluate the translation accuracy and cultural suitability of other apps and in other contexts.

Given how rapidly technology develops and changes, it was not possible to capture every available iPad-compatible app. Therefore, this study provides a snapshot of the available iPad-compatible translation apps and considerations for use in the health care setting. As more apps become available, further research will be required. Furthermore, as this study excluded apps that were available at a cost, only available on Android devices, or involved languages other than English as the primary language, further research evaluating these apps is warranted.

### Conclusions

Overall, the findings of this evaluation have identified that iPad-compatible language translation technology requires careful consideration when used in health care settings, and it may be completely or partly prohibited by existing health care policies. The degree of suitability for health care settings varies on the basis of the content and features available within each app. Those that allow translation of free voice, text, or image information were deemed to be the least suitable for health care settings. Of the apps evaluated, only 2 were considered to be highly suitable for the health care setting, on the basis of their use of preset health related phrases; these were CALD Assist and Talk To Me. When the content was appropriate, preset health phrases were considered the most suitable because information was brief, simple, and contained. Although many apps featured preset phrases, the content was frequently considered unsuitable as phrases were overly complex, lengthy, contained sensitive information, and did not allow for an appropriate answer. When considering the use of translation technology in health care settings, clinicians are encouraged to consider the capabilities of the translation technology itself, as well as the particular situation, the patient, and any organizational policies. Translation technology is not an appropriate substitute for a professional interpreter and further research is required to evaluate its use for everyday conversations in real clinical settings. However, it is not logistically or financially possible to access a professional interpreter for every interaction in a patient’s health care episode. Therefore, there is a pressing need to develop tools that facilitate this communication in a safe and effective manner. Translation technology can play an important role, but this research clearly shows the importance of considering the scope of its use.

## Appendix

Multimedia Appendix 1 Feature analysis stage.

## Abbreviations

CALD: culturally and linguistically diverse
NES: non-English speaking
