# Supplementary material for: Language Translation Apps in Health Care Settings: Expert Opinion
Source: JMIR Mhealth Uhealth. 2019 Apr 9;7(4):e11316. doi: 10.2196/11316 (PMC6477569; doi:10.2196/11316)
Supplement: Multimedia Appendix 1 [file mhealth_v7i4e11316_app1.docx]

| **App name** | **Developer** | **Year** | **Offline use?** | **Function available** | | | | | **Maximum**  **# of supported languages** | **Developer’s use intended for health care settings** | **Comments/ issues identified by the researchers** |
| --- | --- | --- | --- | --- | --- | --- | --- | --- | --- | --- | --- |
|  |  |  |  | *V2V^a^* | *V2T^b^* | *T2V^c^* | *T2T^d^* | *PHP^e^* |  |  |  |
| 1. CALD Assist | CSIRO, Australia | 2016 | ✓ | 🗴 | 🗴 | 🗴 | 🗴 | ✓ | 10 | ✓  Nurses and allied health clinicians to communicate with patients from CALD backgrounds when an interpreter is not available | All languages downloaded with application.  Easy to use.  iPad only. |
| 2. Canopy Speak | Canopy Innovations Inc., United States of America | 2014 | ✓ | 🗴 | 🗴 | 🗴 | 🗴 | ✓ | 15 | ✓  Communicate quickly with patients using translated medical phrases | Can be used offline.  No free input, pre-set phrases only.  Difficult to use and navigate within the topics provided. |
| 3. Dr. Passport – Personal | MAIS Co., Ltd., Japan | 2015 | ✓ | 🗴 | 🗴 | 🗴 | 🗴 | ✓ | 13 | ✓  Allows patients to communicate symptoms to medical professionals in their own language when travelling abroad | Difficult to use.  Some languages require in-app purchases.  Led by patient, not doctor. |
| 4. Google Translate | Google, Inc., United States of America | 2011 | ✓ | ✓ | ✓ | ✓ | ✓ | 🗴 | 103 | 🗴 | Must be online for V2V, V2T and T2V. Only T2T in offline mode.  Languages must be downloaded.  Also enables image translator and writing translator.  Easy to use. |
| 5. iTranslate | iTranslate, Austria | 2008 | 🗴 | ✓ | ✓ | ✓ | ✓ | 🗴 | 34 | 🗴 | Must be online for all functions.  Free 7 days, then $5.99/month USD.  Keeps conversation history. |
| 6.iTranslate VOICE | iTranslate, Austria | 2015 | 🗴 | ✓ | ✓ | 🗴 | 🗴 | 🗴 | 42 | 🗴 | Must be online for all functions.  Free 7 days, then $5.99/month USD.  Can edit text of voice input but cannot directly input text.  Very limited pre-set phrases that may not be suitable to a health care setting. |
| 7. MediBabble Translator | NiteFloat, Inc., United States of America | 2011 | ✓ | 🗴 | 🗴 | 🗴 | 🗴 | ✓ | 5 | ✓  History-taking, initial assessments and physical exam application designed to improve the safety, efficiency, and quality of care for non-English speaking patients. | Can be used offline.  No free input, pre-set phrases only.  Very difficult to use and too much detail. |
| 8. Microsoft Translator | Microsoft Corporation, United States of America | 2015 | ✓ | ✓ | ✓ | ✓ | ✓ | 🗴 | 60 | 🗴 | Must be online for V2V, V2T and T2V. Only T2T in offline mode.  Group conversation translator and image translator option.  Easy to use. |
| 9. Naver Papago Translate | NAVER Corp., Korea | 2016 | 🗴 | ✓ | ✓ | ✓ | ✓ | 🗴 | 6 | 🗴 | Must be online for all functions.  Image translator option. |
| 10. Say Hi Translate | SayHi, United States of America | 2011 | 🗴 | ✓ | ✓ | 🗴 | 🗴 | 🗴 | 90 | 🗴 | Must be online for V2V and V2T.  No free text input.  Easy to use. |
| 11. Speak & Translate | Apalon Apps, Belarus | 2014 | 🗴 | ✓ | ✓ | ✓ | ✓ | 🗴 | 117 | 🗴 | Must be online for V2V and V2T.  3 days free, then $9.99/month USD.  Easy to use. |
| 12. Talk To Me | Datacom, New Zealand /Australia | 2017 | ✓ | 🗴 | 🗴 | 🗴 | 🗴 | ✓ | 7 | ✓  Focusses on key areas of communication with a preliminary focus on elderly CALD patients in sub-acute settings | Pre-set phrases only.  Easy to use. |
| 13.TripLingo | TripLingo, LLC, United States of America | 2011 | ✓ 🗴 | ✓ | ✓ | 🗴 | 🗴 | ✓ | 42 | 🗴 | Must be online for V2V, V2T and image translator.  Difficult to use. |
| 14. Universal Doctor Speaker | Universal Projects and Tools S.L., Spain | 2010 | ✓ | 🗴 | 🗴 | 🗴 | 🗴 | ✓ | 17 | ✓  Facilitates communication between patients and healthcare professionals who don't share a common language | Can be used offline.  No free input, pre-set phrases only. |
| 15. Waygo | Translate Abroad, United States of America | 2012 | ✓ | 🗴 | 🗴 | 🗴 | 🗴 | 🗴 | 4 | 🗴 | Can be used offline.  Image translator only.  Does not allow from English to target language. |
